# On modifications to the Poisson-triggered hidden Markov paradigm through partitioned empirical recurrence rates ratios and its applications to natural hazards monitoring

**DOI:** 10.1038/s41598-020-72803-z

**Published:** 2020-09-28

**Authors:** Moinak Bhaduri

**Affiliations:** Department of Mathematical Sciences, Bentley University, Waltham, MA 02452 United States

**Keywords:** Climate sciences, Environmental sciences, Natural hazards, Mathematics and computing

## Abstract

Hidden Markov models (HMMs), especially those with a Poisson density governing the latent state-dependent emission probabilities, have enjoyed substantial and undeniable success in modeling natural hazards. Classifications among these hazards, induced through quantifiable properties such as varying intensities or geographic proximities, often exist, enabling the creation of an empirical recurrence rates ratio (ERRR), a smoothing statistic that is gradually gaining currency in modeling literature due to its demonstrated ability in unearthing interactions. Embracing these tools, this study puts forth a refreshing monitoring alternative where the unobserved state transition probability matrix in the likelihood of the Poisson based HMM is replaced by the observed transition probabilities of a discretized ERRR. Analyzing examples from Hawaiian volcanic and West Atlantic hurricane interactions, this work illustrates how the discretized ERRR may be interpreted as an observed version of the unobserved hidden Markov chain that generates one of the two interacting processes. Surveying different facets of traditional inference such as global state decoding, hidden state predictions, one-out conditional distributions, and implementing related computational algorithms, we find that the latest proposal estimates the chances of observing a high-risk period, one threatening several hazards, more accurately than its established counterpart. Strongly intuitive and devoid of forbidding technicalities, the new prescription launches a vision of surer forecasts and stands versatile enough to be applicable to other types of hazard monitoring (such as landslides, earthquakes, floods), especially those with meager occurrence probabilities.

## Introduction

Despite differences stemming from assignable factors such as seasonality (hurricanes, unlike earthquakes, for instance, have a tendency of striking around the third quarter), natural calamities share striking similarities—the most unnerving one, perhaps, being their unpredictability. Closer scrutiny often reveals intriguing classifications—hurricanes, as an example, may be categorized into strong and weak types depending on the maximum wind speeds attained, earthquakes into mild and severe, depending on the magnitudes they clocked on the Richter scale, or volcanic eruptions into minor and major, depending on the amount of lava generated or the expanse of area covered. Experts in the respective fields dictate the thresholds defining the partitions (we will follow the National Oceanic and Atmospheric Association’s wind speed-based classification in a later example) and debate on whether alternate requirements may be imposed (such as the number of people killed by a hurricane instead of, or in addition to its maximum wind speed). In spite of such subjectivity, the existence of categorization remains undeniable. This, in turn, enables one to construct the empirical recurrence rates ratio (ERRR), a statistic introduced by Ho et al.^1^, and popularized by Ho and Bhaduri^2^, Bhaduri and Ho^3^ that reflects the interaction between two such groups. A brief reminder will be provided in the next section on the way this function is defined and the way it operates.

One-step Markov chains, on the other hand, are apt tools to model random phenomena where the state of a system at any given time depends probabilistically on only the one at the previous time instant and not on the rest of its history. The requirement will be mathematically formalized in the section below on our methodology. Intricacies and variants surely are in vogue, and we direct interested readers to Daley^4^, Zhan et al.^5^, Koutras^6^, for instance. Such chains have found applications even in movement research^7^. Furthermore, if it can be assumed (and we will implement tests confirming this) that the observations tracking the series of interest are generated by probability distributions relying on some underlying *latent* states, and that the state space is equipped with the one-step Markov property described previously, the resulting framework is known as a hidden Markov model (HMM). HMMs have their roots in a series of papers written by Baum and colleagues^8–10^. Additionally, Ghahramani^11^ offers an excellent introduction to the topic. These HMMs have proven themselves useful in modeling series that are over-dispersed, in handwriting recognition systems^12^, gesture recognition under variation^13^, sentence lipreading^14^, tracking and surveillance in spatial environments^15^, shape tracking and production^16^, image retrieval of rotated objects^17^, bioinformatics^18^, among others. In more recent times, HMMs are being deployed in rare event detection^19^ and anomaly detection (through a slightly generalized version of self-adaptive HMMs) for behavior recognition^20^ and drift detection in computer science^21,22^. Macdonald and Raubenheimer^23^ have used HMMs to analyze the locomotory behavior of locusts. Extensions, demonstrated by Mutschler and Philippsen^24^, are possible to formulate noisy versions of these, termed nHMMs. Other generalizations of HMMs (pairwise and triplet models) are now used to model various kinds of non-stationary data, surveyed by Lanchantin and Pieczynski^25^ and Boudaren et al.^26^.

The purpose of this work is to fuse these two notions of ERRRs and HMMs through a uniquely novel claim—once accurately discretized, the ERRR series may be taken as an *observed* version of the unobservable state space generating the count series of one of the two processes involved. The resulting inferences (including forecasts) will be considerably more accurate than those from the traditional HMM setup, in which these hidden states can only be estimated, but never observed. The paper is organized through several sections. The next introduces the two data sets we intend to examine, and for the sake of completeness, we will briefly explain the ERRR statistic introduced in some of our previous works, here. The third section describes the mathematical preliminaries, along with our proposal on modifying the established Poisson-based HMM. The fourth, through its several subsections, will survey various aspects of statistical inference along with recording the benefits that ensue from implementing the modification. The last contains some concluding observations and remarks on how this work may prosper.

## Data sets and empirical recurrence rates ratio

To demonstrate the applicability of our proposal, we have sampled two data sets, one from volcanology, the other from weather science, one equipped with a seasonal structure, the other not. Both enjoy the luxury of moderately rich histories, one worth 90 years’, while the other 236 years’. We are confident that due to Empirical Recurrence Rates’ (ERRs’, a building block of ERRR, described below) demonstrated ability^27,28^ to model rare events without such rich past, the method we are going to develop should find wider applicability.

### Volcanism

The United States Geological Survey (at https://volcanoes.usgs.gov/index.html ) and the Smithsonian Institution’s Global Volcanism Program (at https://volcano.si.edu/) maintain extensive records of volcanic eruptions worldwide, including the duration of activity, the amount of lava generated, the area covered, the data collection method employed, etc. We have collected the eruption times of two Hawaiian volcanoes—Kilauea and its close neighbor Mauna Loa from these sources, partly because of their relevance (at this writing, Kilauea has grown increasingly restless, displacing scores of inhabitants from its vicinity) and partly because of their prominence in geoscience literature. Eruption patterns of these two have been studied by Lipman^29^, Klein^30^, and several others, notably Miklius and Cervelli^31^ and Walter and Amelung^32^, in more recent times. We point readers to Ho and Bhaduri^2^ for a more extensive literature review and the 63 and 40-length time series storing the dates of Kilauea and Mauna Loa eruptions respectively, over the period 1750–1985.

### Oceanography

Our next example concerns West Atlantic hurricanes, another menace that torments the US east coast with annoying regularity. The Hurricane Center of the National Oceanic and Atmospheric Administration (NOAA) (at https://www.nhc.noaa.gov/?cpac), under the US Department of Commerce, is a significant authority in this regard, tracking storms all over the globe along with pertinent details like their speeds, estimated routes, etc. Classification, in the sense described in the previous introductory section, is done through the maximum wind speeds attained (Table 1).

**Table 1:** NOAA hurricane classification through maximum wind speeds.

Category	Maximum wind speed
Hurricane 5	$>135$ knots
Hurricane 4	114–135 knots
Hurricane 3	96–113 knots
Hurricane 2	83–95 knots
Hurricane 1	64–82 knots
Trop/subtropical storm	34–63 knots

Following leading experts such as Emanuel^33–35^, we have treated category 5, 4, and 3 hurricanes as the *strong* ones, 2 and 1 hurricanes as the *weak* ones, and the rest as the *tropicals*. Over the period 1923–2013, we found records on 32 H5, 84 H4, 87 H3, 93 H2, 150 H1, 271 tropical and 24 subtropical storms. A comparison among these has been carried out by Bhaduri and Ho^3^.

### Empirical recurrence rates ratio (ERRR)

Corresponding to a discrete time series $\{ X_{i}\}_{i=1,2,\ldots }$ that counts the number of times an event happens over $(i-1,i]$ units, the Empirical Recurrence Rate (ERR) statistic at time *t* is defined as

1 $$\begin{aligned} Z_{t}=\frac{\sum _{i=1}^{t}X_{i}}{t}, \ \ t=1,2,\ldots \end{aligned}$$

Introduced in the context of rare event modeling by Tan, Bhaduri, and Ho^27^and Ho and Bhaduri^28^, through the process of cumulating histories, ERR generates non-zero observations over barren periods of the original time series, thereby making the modeling of zero-inflated processes more tractable. In case the events can be modeled through a homogeneous Poisson process, ERR will track the maximum likelihood estimate of the underlying rate of occurrence. Time series modeling of this quantity has found success in strong sandstorm (seasonality present) and earthquake (seasonality absent) prediction, evidenced through some of our recent works^27,28^. ERR was next generalized to empirical recurrence rates ratio (ERRR) by Ho et al.^1^, Ho and Bhaduri^2^, Bhaduri and Zhan^36^, Bhaduri and Ho^3^, and Zhan et al.^5^ and the latter operates, through the presence of another, possibly related series $\{ Y_{i}\}_{i=1,2,\ldots }$ as

2 $$\begin{aligned} R_{t}=\frac{\sum _{i=1}^{t}X_{i}}{\sum _{i=1}^{t}X_{i}+\sum _{i=1}^{t}Y_{i}}, \ \ t=1,2,\ldots \end{aligned}$$

**Figure 1:**
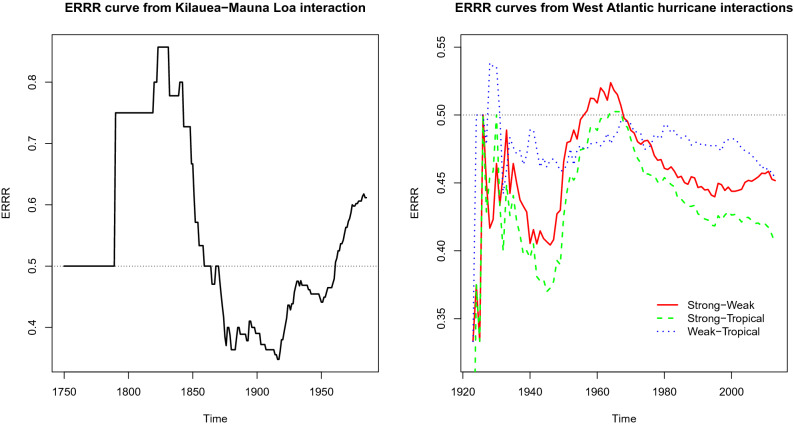
ERRR curves from volcanic (left panel) and hurricane (right panel) interactions.

Dividing both numerator and denominator by the time length *t*, one may express ERRR indeed as the ratio of two ERRs. Once plotted as a function of time, ERRR reveals interesting patterns (Fig. 1).

Ho et al.^1^, Ho and Bhaduri^2^, Bhaduri and Zhan^36^ and Bhaduri and Ho^3^ demonstrate that if the $(X_{i},Y_{i})_{i=1,2,\ldots }$ observations are inversely related, i.e., if one’s activity goes hand-in-hand with the other’s dormancy, the resulting ERRR curve will exhibit wave-like properties, else a monotonic trend. Using techniques such as block bootstrapping, Ho and Bhaduri^2^ proposed measures like the *index of waviness* to quantify sinusoidal tendencies, promising a few more in the offing. This work found that if the $\{ X_{t}\}_{t=1,2,\ldots }$ series is taken as the Kilauea eruption counts and $\{ Y_{t}\}_{t=1,2,\ldots }$ and the one for Mauna Loa, the resulting ERRR curve, graphed in the left panel of Fig. 1 will be considerably wavy. This hints at a possible inverse dependence between the two volcanoes lending credence to such a possibility held by Lipman^29^ and others. An explanation was offered through the possible connectedness of their magma reservoirs, making one active over a time period on which the other one is relatively dormant.

Similar interaction conjectures abound with hurricane frequencies too. Weather scientists are of the opinion^33–35^ that as the ocean temperature rises, the likelihood of a storm creation drops due to an increase in saturation deficit. However, if one gets started somehow, it has the potential to become deadly. Thus, despite a drop in the overall hurricane count, the proportion of strong ones should rise in recent times. Using the ERRR statistic, Bhaduri and Ho^3^ have shed light on this issue. With the strong–weak-tropical classification, $3 \atopwithdelims ()2 =3$ pairs may be formed, with each generating one ERRR curve. The right panel of Fig. 1 depicts all on the same graph, with the strong–weak curve, for instance, created by treating the strong hurricane counts as the $\{ X_{t}\}_{t=1,2,\ldots }$ series and the weak counts as the $\{ Y_{t}\}_{t=1,2,\ldots }$ series. They all exhibit oscillatory tendencies, with the strong–weak curve indeed on the rise, indicating an increased proportion of devastating hurricanes. Interestingly, these three curves intersect around 1970, a time since when global warming tightened its grip.

Thus we observe the ERRR statistic can be used to glean insights into the dependence structure between two series. In the hurricane example, these two were a product of classification (described in the introduction), while in the volcanic example, these two were guided by geographic proximity. By construction, the ERRR curve remains bounded by 0 and 1. This is because we are tacitly dealing with non-negative counts, and agree to remove from ERRR construction any possible initial period where both the contributing series are entirely silent, triggering a zero denominator for the fraction (2). In the following sections, we will break up this [0, 1] range into disjoint blocks to construct a discrete chain that mimics the latent states in an HMM.

## Methodology

The first mathematical concept needed for a firm understanding of HMM, and hence, of our proposal, is the one of (ordinary, as opposed to hidden) Markov chains. A phenomenon $\{ C_{t}\}_{t=1,2,\ldots }$ observed at discrete time points $t=1,2,\ldots$ is said to follow the Markov property, and hence, constitute a Markov chain if

3 $$\begin{aligned} P\{ C_{t}=c_{t}|C_{t-1}=c_{t-1}, C_{t-2}=c_{t-2}, \ldots , C_{1}=c_{1}\} = P\{ C_{t}=c_{t}|C_{t-1}=c_{t-1}\}, \end{aligned}$$

i.e., the probabilistic knowledge about its state at any given time draws only from the preceding time instant, and not from the rest of its history. A matrix $\Gamma = ((\gamma _{ij}))$, termed the transition probability matrix (t.p.m), stores $\gamma _{ij}$s, the probabilities of a switch from state *i* to state *j*. These, in general, are unknown parameters, and may be estimated once the chain is observed as $\hat{\gamma _{ij}}=\frac{n_{ij}}{n_{i+}}$, where the numerator $n_{ij}$ represents the total number of such transitions and the denominator $n_{i+}$ represents the total number of transitions out of state *i*. If a probability distribution $\vec {\pi }$ (on the state space) exists such that $\vec {\pi }\Gamma = \vec {\pi }$ holds, $\vec {\pi }$ is called the stationary distribution of the chain, i.e., regardless of the chain’s starting state, in the long run, the proportion of times it will spend in state *k* is $\pi _{k}$. The one step memory (3), for varied reasons, has been generalized to $s>1$ steps, and the discrete time framework to continuous time^37^. For our purpose, however, (3) will be apt and now we proceed to elaborate on Poisson-HMM, an established method to model rare events, and ERRR-HMM, our recommendation on modifying it.

### Poisson-HMM

The use of HMMs, especially the Poisson-based ones we are going to improve on, in natural hazards monitoring has grown profuse in recent times. Through a dynamic incorporation of spatial and temporal components, Wang et al.^38^ have used HMMs to model risk assessments of rainstorms in the Chinese province of Dalian. Khadr^39^ has employed an array of HMMs to forecast droughts in the upper Blue Nile river basin. The method has been shown to be efficient in sounding early drought warnings and differentiating events (i.e., hazards) from non-events. Wu^40^ formulated a simple HMM occurrence model for earthquake de-clustering using data from central and western Japan. Wu^41^ developed a larger class of models, termed quasi-HMMs, generalizing usual HMMs, to estimate the most likely location of an earthquake aftershock in central New Zealand. Joshi et al.^42^ used HMMs for avalanche danger simulations on road sectors in North-West Himalayas. In recent times, as evidenced by Zhu et al.^43^, HMMs are finding applications in modeling unreliable communication channels through an interesting class of Lyapunov functions.

HMMs essentially represent a type of two-tier hierarchical modeling where one tier, the observations that an experimenter sees (represented by *X*s), is observed, and the other, the states of an underlying Markov chain that generates them (represented by *C*s) remains unobserved. With the random vectors $\vec {X}^{(t)}:=(X_{1},X_{2},\ldots ,X_{t})$ and $\vec {C}^{(t)}:=(C_{1},C_{2},\ldots ,C_{t})$ tracking the histories of the observations and the hidden chain, respectively, a compact shorthand of a HMM is given through

4 $$\begin{aligned}&P(C_{t}|{\vec {C}}^{t-1})=P(C_{t}|C_{t-1}), t=2,3\ldots \end{aligned}$$

5 $$\begin{aligned}&P(X_{t}|{\vec {X}}^{t-1},{\vec {C}}^{t})=P(X_{t}|C_{t}), t \in N. \end{aligned}$$

The Markov property (3), seen here through (4), therefore, holds at the level of the latent states. The observations rely on the states in a specific way—given the present state $C_{t}$, the probability distribution of $X_{t}$ is independent of the past observations or states. If the goal is to model discrete, non-negative counts, it is often convenient to require the state-dependent observation density to have a parametric form such as the Poisson, i.e., $\forall x= 0,1,2,\ldots , i=1,2,\ldots ,m$,

6 $$\begin{aligned} p_{i} (x) = P(X_{t} = x| C_{t} = i) = e^{-\lambda _{i}}\frac{\lambda _{i}^{x}}{x!}, \end{aligned}$$

giving the underlying chain *m* states to move on, leading to an *m*-state Poisson-HMM. Several authors have used this setup, captured through (4), (5) and (6), to track longitudinal and spatial data and to resolve classification problems. Zucchini and MacDonald^44^, for instance, have shown how the Poisson-HMM can incorporate temporal dependence and over-dispersed models while tracking a time series storing earthquake counts. At any given instant, one of *m* Poisson distributions with means $\lambda _{1},\lambda _{2},\ldots , \lambda _{m}$ was thought to remain active. This distribution generated the count at that time through (6) where the $\lambda$ value gets chosen according to a further random tool, the parameter process—if $\vec {\delta }:=(\delta _{1},\delta _{2},\ldots ,\delta _{m})$ represent the stationary distribution of the t.p.m. of the underlying Markov chain, the mean $\lambda _{i}$ is chosen with probability $\delta _{i}$. The likelihood of observing $x_{1},x_{2},\ldots ,x_{t}$ as the count sequence is given by

7 $$\begin{aligned} L_{T} = \vec {\delta }P(x_{1})\Gamma P(x_{2})\ldots \Gamma P(x_{T})1^{'} \end{aligned}$$

where $\vec {\delta }$ is the initial distribution of the chain, and $\Gamma$ is its t.p.m. Here *P*, given by

$$\begin{aligned} \left( \begin{array}{cccc} p_{1}(x) &{} 0 &{} ... &{} 0 \\ 0 &{} p_{2}(x) &{} ... &{} 0 \\ ...&{}... &{} ... &{} ... \\ 0 &{} 0 &{} ... &{} p_{m}(x) \end{array} \right) \end{aligned}$$

is a $m \times m$ diagonal matrix storing the state-dependent probabilities in (6). If the initial distribution $\vec {\delta }$, (i.e., the probabilistic law of $C_{1}$) also serves as the chain’s stationary distribution, then (7) changes to

8 $$\begin{aligned} L_{T} = \vec {\delta }\Gamma P(x_{1})\Gamma P(x_{2})\ldots \Gamma P(x_{T})1^{'}. \end{aligned}$$

We notice that the parameters that have to be estimated using (7) or (8) from the data are only the components of $\Gamma$ and the mean vector $\vec {\lambda }$. This is because $\vec {\delta }$ may be found from

9 $$\begin{aligned} \vec {\delta }(I_{m} - \Gamma + U) = 1^{'} \end{aligned}$$

where $1^{'}$ is a vector of ones and *U* is an $m \times m$ matrix of ones. The maximum likelihood estimation can be done, as shown by Zucchini and MacDonald^44^ either through maximizing (7) or (8) directly or through the Expectation–Maximizing (E–M) algorithm. We shall denote the estimates obtained this way by $\hat{\vec {\lambda }}$ and ${\hat{\Gamma }}_{Pois-HMM}$.

The earliest applications of Poisson-HMMs date back to the early 1900s with scholars such as Albert^45^, Leroux^46^, Le et al.^47^, Leroux and Puterman^48^ developing it to study time series of epileptic seizure counts. In more recent times, Can et al.^49^ used Poisson-HMMs to estimate earthquake (magnitude four or higher on the Richter scale) hazards over the period 2013–2047 in north-western Turkey. Orfanogiannaki et al.^50^ applied the Poisson-HMM to model earthquake frequencies in the seismogenic area of Killini, Ionian Sea, Greece, between the period 1990 and 2006. Mendoza-Rosas and la Cruz-Reyna^51^ used a mixture of exponential densities (the distribution of inter-event times in case the events may be modeled by a stationary Poisson process) to examine the repose times of the Colima and Popocatepetl volcanoes in Mexico. Bebbington^52^ has used Poisson based HMMs to locate eruption regimes of volcanoes using data on Mt. Etna from 1600 to 2006.

### ERRR-HMM

In case the number of latent states is *m*, the Poisson-HMM framework, elaborated in the previous subsection, requires the estimation of $m+(m-1)\times m = m^{2}$ unknown parameters. This is because it involves *m*-many $\lambda$ values, and an *m*-dimensional square t.p.m. $\Gamma$, in which, the number of free parameters is $(m-1)\times m$ (the last entry in any row can be calculated using the fact that the row sums must equal unity, since the chain, at the next instant, *must* move to some state, including quite possibly, where it already was). Furthermore, as we shall see, a Poisson-HMM furnishes only an *estimate* of the most likely states of the hidden chain. In case additional relevant information (like the Mauna Loa eruption or the weak hurricane counts) that is likely to influence the target series of interest (the Kilauea eruption or the strong hurricane counts) become readily available, we propose to use a discretized version of the generated ERRR series as a chain triggering the observations. This chain, unlike the one from the traditional Poisson-HMM, is easily calculable from the data values $\{ X_{i}\}_{i=1,2,\ldots }$ and $\{ Y_{i}\}_{i=1,2,\ldots }$ themselves, and hence, will be observed. Estimation of the t.p.m.s in (7) and (8) will not involve the E–M algorithm mentioned in the previous section or similar technical tools, but rely instead on simple relative frequency based non-parametric methods described in the previous subsection. Although this matrix estimation problem persists, the computational and the conceptual complexity drops considerably, requiring now only the guessing of *m*-many $\lambda$ values. Our proposal is thus, seemingly parsimonious. While such remains the rationale behind and the broad overview of ERRR-HMM, regarding its implementation, we offer two alternatives.

One could plug in the t.p.m. estimate ${\hat{\Gamma }}_{ERRR-HMM}$ in (7) or (8) and maximize these to get the maximum likelihood estimates of $\vec {\lambda }$. However, we found that depending on the data being analyzed, this might lead to numerical over/underflow. The remedy could be reparametrization, or our second alternative—we would go through the usual Poisson-HMM workflow and at the end, would replace ${\hat{\Gamma }}_{Pois-HMM}$ by ${\hat{\Gamma }}_{ERRR-HMM}$, with $\vec {\lambda }$ remaining unchanged. Arguably, this is a sacrifice on the parsimony of ERRR-HMM. Despite that, in the next section, we will implement this second option on the two data sets described previously and demonstrate its superiority over the established Poisson-HMM.

## Results

The count-based examples we have sampled from volcanology and weather science serve as proper instances on which to implement both the Poisson-HMM and ERRR-HMM techniques. This is because our intention is to model non-negative counts that are unbounded with possibly decaying probabilities, for which, the Poisson density expressed through (6) is an apt choice. In addition, as evidenced by Zucchini and MacDonald^44^, Poisson-HMMs can tackle overdispersion issues in the $\{ X_{i}\}_{i=1,2,\ldots }$ values which plague the Kilauea count series (we found a mean of 0.267 and a variance of 0.401).

### Chain creation and validation

This section describes the ERRR-discretization mentioned in the previous ones along with statistical tests to ensure such discretized ERRRs may be treated as Markov chains. We partition the volcanic ERRR range (graphed in the left panel of Fig. 1) into the following states:

$$\begin{aligned} c(t) = {\left\{ \begin{array}{ll} 1 &{} \text {if }\, r_{t} \in [0,0.4) \\ 2 &{} \text {if } \, r_{t} \in [0.4,0.6) \\ 3 &{} \text {if }\,  r_{t} \in [0.6,0.8) \\ 4 &{} \text {if } \, r_{t} \in [0.8,1] \end{array}\right. } \end{aligned}$$

We will elaborate on the reasoning behind four partitions in the subsection to follow. For now, we check, given its history, whether this discretized ERRR $\{ C_{t}\}_{t=1,2,3,\ldots }$ may be treated as a Markov chain.

While choosing one of the two hypotheses

10 $$\begin{aligned}&H_{0}: C_{1},C_{2},\ldots ,C_{T}|c_{0} \ \text {are independent} \end{aligned}$$

11 $$\begin{aligned}&H_{a}: C_{1},C_{2},\ldots ,C_{T}|c_{0} \ \text {is a Markov Chain with unknown t.p.m} \Gamma = ((\gamma _{ij})) \end{aligned}$$

it may be shown that the conventional test statistic $U= 2 \sum \nolimits _{i=1}^ m \sum \nolimits _{i=1}^m n_{ij} \frac{{\hat{\gamma }}_{ij}}{{\hat{\gamma }}_{j}}$ follows a chi-square distribution with $(m-1)^{2}$ degrees of freedom under the null assumption of independence, where *m* represents the number of hidden states, $n_{ij}$, the number of transitions from state *i* to *j*, ${\hat{\gamma }}_{ij}=\frac{n_{ij}}{n_{i+}}$, the estimated probability of this transition ($n_{i+}=\sum _{j=1}^{m}n_{ij}$). For the present volcanic example, $m=4$ and $U = 206.822$ with the upper 10%, 5%, and 1% chi-square cutoffs given by $\chi _{9,0.1}^{2}=14.68, \chi _{9,0.05}^{2}=16.92, \chi _{9,0.01}^{2}=21.67$, respectively. Thus, with the observed statistic exceeding these thresholds at these usual levels of significance, we may take $\{ C_{t}\}_{t=1,2,\ldots }$ as a discrete-time, discrete-space Markov chain. The estimates of the transition probabilities given by ${\hat{\gamma }}_{ij}=\frac{n_{ij}}{n_{i+}}$ are contained in

$$\begin{aligned} {\hat{\Gamma }} = \left( \begin{array}{cccc} 0.9 &{} 0.1 &{} 0 &{} 0 \\ 0.032 &{} 0.952 &{} 0.016 &{} 0 \\ 0 &{} 0.0169 &{} 0.949 &{} 0.034 \\ 0 &{} 0 &{} 0.182 &{} 0.818 \end{array} \right) . \end{aligned}$$

From the second example on hurricane counts graphed on the right panel of Fig. 1, we have isolated the “Strong–Weak” ERRR curve and discretized it (reasons explained in the next subsection) according to

$$\begin{aligned} c(t) = {\left\{ \begin{array}{ll} 1 &{} \text {if } \, r_{t} \in [0,0.45) \\ 2 &{} \text {if }\,  r_{t} \in [0.45,1] \end{array}\right. } \end{aligned}$$

The estimated transition probabilities from this example are stored through

$$\begin{aligned} {\hat{\Gamma }} = \left( \begin{array}{cc} 0.911 &{} 0.089 \\ 0.147 &{} 0.853 \end{array} \right) . \end{aligned}$$

### Choice of the number of hidden states

The number *m* of states that the hidden Markov chain may flow over is, in general, unknown, and care must be exercised in its estimation. There remain a few options, one of which involves imposing Bayesian priors on this quantity and comparing the posterior odds. This might, however, raise concerns about the subjectivity in the choice of prior distributions. We therefore, will adopt a different alternate—estimate *m* through the smallest Akaike Information Criteria (AIC) or Bayesian Information Criteria (BIC). Both Poisson-HMMs and ERRR-HMMs may be fitted infinitely accurately by inflating the number of hidden states *m*, essentially increasing the number of free parameters, and hence, complicating the model, much akin to the overfitting problem in regression. AIC and BIC are designed to prevent such tendencies. Usually written in the form

12 $$\begin{aligned} -2\log L +kp, \end{aligned}$$

where *L* is the likelihood and *p* is the number of parameters, these quantities penalize complex model-generated likelihoods through the positive second term. The value of *k* is set at 2 for AIC and $\log n$ (where *n* is the sample size) for BIC and further details about their workings and relative merits may be had from Akaike^53^ and Wit and den Heuvel^54^. Smaller values of AIC or BIC are generally preferred.

We varied *m* over several values for both our examples and Tables 2 and 3 record the resulting changes in the log-likelihood and AIC and BIC values.

**Table 2:** Optimum number of hidden states, hurricane interaction.

*m*	$-\log Lik$	AIC	BIC
2	184.85	377.72	387.76
3	184.30	386.60	409.20
4	183.81	399.61	439.79

**Table 3:** Optimum number of hidden states, volcanic interaction.

*m*	$-\log Lik$	AIC	BIC
2	133.38	274.76	288.62
3	133.24	284.47	315.65
4	126.08	284.17	339.59

On the hurricane example (Table 2), we find that the AIC and BIC values are minimized for a simple two-state model. This explains using $m=2$ in later sections and partitioning the Strong–Weak ERRR curve into two states in the previous subsection. With the volcanic example (Table 3), however, despite $m=2$ achieving the smallest AIC, BIC values again, we opt for a slightly complex four-state model. This is because unlike the hurricane example, the decrease in the negative log-likelihood value from $m=2$ to $m=4$ in this case, is quite significant.

### The estimates

A broad overview of the estimation procedures involved in both the established Poisson-HMM and our ERRR-HMM proposal has been described in section 3***. Our numerical maximization of (7) and (8) proceeded along lines described by Zuchchini and MacDonald^44^, using reparametrization, choosing the observed quantiles as the seed values for $\lambda _{i}$s, and using off-diagonal seeds of 0.05 in $\Gamma$. The maximum likelihood estimates under the Poisson-HMM framework for the volcanic interaction case are

13 $$\begin{aligned} \hat{\lambda _{1}}= & {} 3.49 \times 10^{-69}, \hat{\lambda _{2}} = 1.03 \times 10^{-32}, \hat{\lambda _{3}} = 0.8, \hat{\lambda _{4}} = 0.923, \nonumber \\ {\hat{\Gamma }}_{P-HMM}= & {} \left( \begin{array}{cccc} 0.87 &{} 0.11 &{} 7.79 \times 10^{-57} &{} 0.01 \\ 6.39 \times 10^{-7} &{} 0.19 &{} 0.81 &{} 1.92 \times 10^{-25} \\ 0.83 &{} 1.2 \times 10^{-81} &{} 0.166 &{} 4.24 \times 10^{-13} \\ 1.02 \times 10^{-111} &{} 0.003 &{} 1.94 \times 10^{-113} &{} 0.96 \end{array} \right) . \end{aligned}$$

Following our proposed estimation outline described in section 3, the mean estimates under the ERRR-HMM setup remains unchanged. The state-transition probabilities, estimated through relative frequencies, as seen before, is

$$\begin{aligned} {\hat{\Gamma }}_{ERRR-HMM} = \left( \begin{array}{cccc} 0.9 &{} 0.1 &{} 0 &{} 0 \\ 0.032 &{} 0.952 &{} 0.016 &{} 0 \\ 0 &{} 0.0169 &{} 0.949 &{} 0.034 \\ 0 &{} 0 &{} 0.182 &{} 0.818 \end{array} \right) . \end{aligned}$$

The negative log-likelihood value for the Poisson-HMM setup was 126.08, evidenced also through Table 3 (with $m=4$), while the value for the ERRR-HMM setup was 150.057. Despite such difference, which could have been made more minimal had we chosen the option $m=2$ for the volcanic example too, forecasts extracted from the new ERRR-HMM approach, as we shall see later, will be more accurate. This is emblematic of similar situations in modeling where a complicated model fits the available data accurately, but predicts new observations poorer than a simpler model that fits the available data slightly worse. For the West Atlantic Strong–Weak hurricane interaction, the estimates are

14 $$\begin{aligned} \hat{\lambda _{1}}= & {} 2.571, \hat{\lambda _{2}} = 4.874, \nonumber \\ {\hat{\Gamma }}_{P-HMM}= & {} \left( \begin{array}{cc} 0.929 &{} 0.071 \\ 0.184 &{} 0.816 \end{array} \right) , \nonumber \\ {\hat{\Gamma }}_{ERRR-HMM}= & {} \left( \begin{array}{cc} 0.911 &{} 0.089 \\ 0.147 &{} 0.853 \end{array} \right) . \end{aligned}$$

It is interesting to note the closeness of the matrices ${\hat{\Gamma }}_{Pois-HMM}$ and ${\hat{\Gamma }}_{ERRR-HMM}$ in both examples. With the $2\times 2$ matrix for the hurricane interaction, the closeness might be alleged to be coincidental. This is another reason for choosing four states for the other example on volcanoes, one that demonstrates fair agreement between both t.p.m.s over most of the sixteen spots (as opposed to only four). Such similarities between the cell probabilities from the two methods generates the first indication that the transition behavior of the discretized ERRR closely resembles the one of the hidden Markov chain the generates the counts of interest—Kilauea eruptions and strong hurricane numbers in the two examples considered.

### Random firings

It follows from the definition of conditional probability $P(C_{t}=i)P(X_{t}=x|C_{t}=i)=P(X_{t}=x, C_{t}=i)$ and the principle of marginalization that the unconditional distribution of the observation sequence $\{ X_{t}\}$ at any given time *t* is given by

15 $$\begin{aligned} P(X_{t}=x)=\sum _{i=1}^{m} P(C_{t}=i)P(X_{t}=x|C_{t}=i), \ \ x=0,1,2,\ldots . \end{aligned}$$

**Figure 2:**
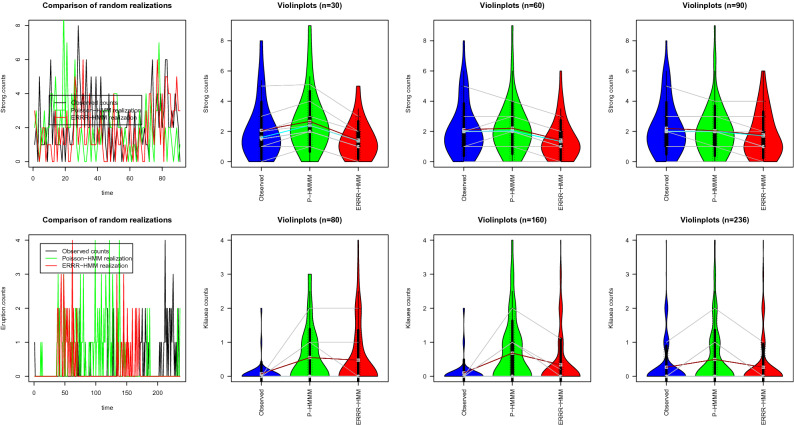
Simulations from hurricane (upper panel) and volcanic (lower panel) interactions.

In Fig. 2, we have simulated random observations from the distribution (15), for both Poisson-HMM and ERRR-HMM with different time blocks of varying lengths, and compared them with the sequence observed in reality.

The leading graphs in both rows demonstrate how the range of values generated by ERRR-HMM is summarily consistent with those from the Poisson-HMM and the observed series. Sets of three violinplots for each case condense summary information (such as medians, boxplots, connected deciles) on the distributions of these simulated values, and describe the effect of longer sequences on the performance of both models (this being another reason behind choosing two examples of such different lengths). We observe that if a process is allowed to propagate for a longer time duration, the distribution of the generated count sequence from ERRR-HMM resembles the actual better than that from Poisson-HMM. This is perhaps most vividly apparent in the second panel on Kilauea eruptions where the ERRR-HMM generated distributions converge faster to the actual than the one given by Poisson-HMM as the sample size increases through rough increments of 80 years.

### Global decoding

The fact that the t.p.m.s of two chains are the same does not imply the chains themselves are identical, viewed temporally. Thus, with the similarity of Poisson-HMM and ERRR-HMM based t.p.ms established in the previous section, this subsection seeks to investigate the similarity between the discretized ERRR and the Poisson-based hidden Markov chain as they evolve in time, through the process of *global decoding*. The method consists of using the observed history $\vec {X_{t}}$ to generate the most likely sequence of hidden states $\vec {C_{t}}$. Mathematically thus, we seek those $c_{1},c_{2},\ldots ,c_{t}$ that maximizes the conditional probability

16 $$\begin{aligned} P(\vec {C^{(T)}} = \vec {c^{(T)}}| \vec {X^{(T)}} = \vec {x^{(T)}}) \end{aligned}$$

The Viterbi algorithm^55^ provides an answer by defining

17 $$\begin{aligned} \epsilon _{1i}= P(C_{1}=i, X_{1}= x_{1}) = \delta _{i} p_{i} (x), \end{aligned}$$

and for $$t=2,3,\ldots ,T$$,

18 $$\begin{aligned} \epsilon _{ti}= \max _{c_{1},\ldots c_{t-1}} P(\vec {C^{(t-1)}} = \vec {c^{(t-1)}}, C_{t} = i, \vec {X^{(T)}} = \vec {x^{(T)}}). \end{aligned}$$

Zucchini and MacDonald^44^ show that for $t=2,3,\ldots ,T$ and $i=1,2,\ldots ,m$, the later simplifies to:

19 $$\begin{aligned} \epsilon _{tj}=(\max _{i} (\epsilon _{t-1,i} \gamma _{ij}))p_{j}(x_{t}) \end{aligned}$$

where $\gamma _{ij}$ as usual, is the (*i*, *j*)th element of the t.p.m. The required maximizing sequence of states $i_{i},i_{2},\ldots ,i_{T}$ can be found through the recursion:

20 $$\begin{aligned} i_{T}=argmax_{i=1,\ldots ,m} \epsilon _{Ti} \end{aligned}$$

and for $t=T-1,T-2,\ldots ,1$ from:

21 $$\begin{aligned} i_{t}=argmax_{i=1,\ldots ,m} (\epsilon _{ti} \gamma _{i,i_{t+1}}) \end{aligned}$$

**Figure 3:**
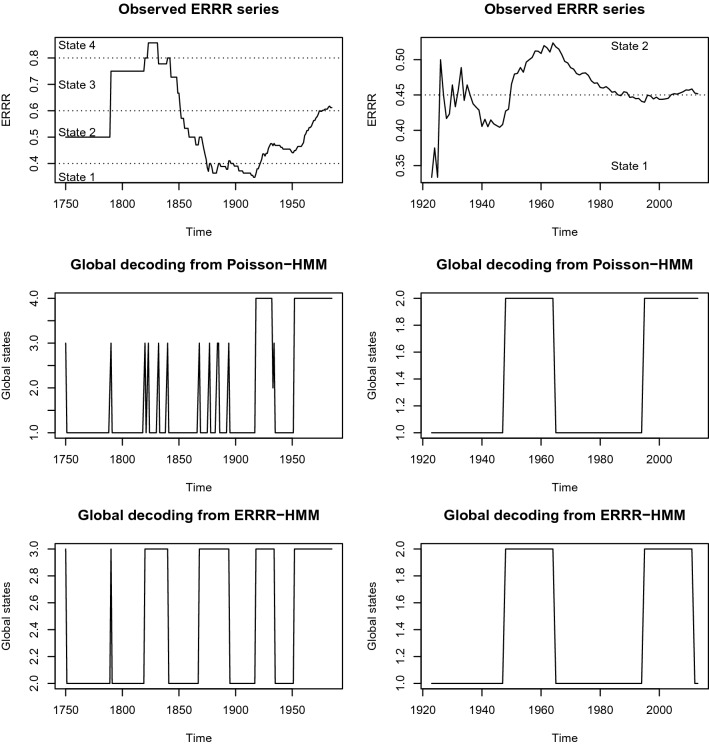
Global deciding from volcanic (left panel) and hurricane (right panel) interactions.

We have applied the Viterbi algorithm with $\gamma _{ij}$s as the estimated transition probabilities found in the previous subsection. The chain sequences are graphed in Fig. 3.

The second rows in both panels depict the most likely way the underlying hidden Markov chain would have evolved through the Poisson-HMM machinery in the absence of additional information about a companion series (Mauna Loa counts for Kilauea and the weak hurricane counts for the strong ones) needed to create ERRR, while the third rows show the evolution in the presence of such information. The first rows re-demonstrate the ERRR curves for easy comparisons. This ERRR curve, we note, is observable, devoid of estimation routines, and free of related uncertainties, relying solely on available data values through (2). The partitions proposed while creating the chain have been overlaid for ready reference. To elaborate, around 1950, using Poisson-HMM, we expect the most likely hidden state of the chain generating the Kilauea counts to be 1. The ERRR-HMM estimates this to be 2, which also happens to be the state at which the discretized ERRR stays at that time. The remarkable proximity, especially in terms of the ongoing trend, between the discretized states of ERRR and the likely chain states extracted from both models, Poisson-HMM and ERRR-HMM, increases confidence in the former generating the Kilauea and strong hurricane counts. The estimated states from the ERRR-HMM (third row), which has information about ERRR embedded in it resembles the discretized states of ERRR (first row) better than those from the Poisson-HMM (second row). Nonetheless, these two competing models generate likely states (second and third rows) that are roughly similar to each other.

### State prediction

Our proposal, ERRR-HMM, can be compared to its traditional counterpart Poisson-HMM through the states they forecast for the near future. In contrast to the predicted counts (i.e., the *number* of Kilauea eruptions or strong hurricanes), which will be elaborated in the next subsection, the witnessed history $\vec {X_{t}}$ may be used to e*xtract state forecasts through

22 $$\begin{aligned} P(C_{T+h} = i| \vec {X^{(T)}} = \vec {x^{(T)}})= \vec {\alpha }_{T} \Gamma ^{h}(,i)/L_{T}, \ \ i=1,2,\ldots ,m, \end{aligned}$$

where *h* is the forecast horizon and $\vec {\alpha }_{t} = \delta P(x_{1}) \prod \limits _{s=2}^t \Gamma P(x_{s})$ and $\Gamma$ is the true transition probability matrix of the hidden chain and $\Gamma ^{h}(,i)$ is the i-th column of $\Gamma ^{h}$, its *h*-fold product. For every value of *h*, thus, (22) generates a probability profile on the space of possible states. To demonstrate, we have set $h=1,100$ and 500 on our volcanic example and have compared the two models through Tables 4 and 5.

**Table 4:** Probability profile on state space from Poisson-HMM, volcanic case.

*m*	$h=1$	$h=100$	$h=500$
1	0.137	0.574	0.577
2	0.057	0.091	0.091
3	0.100	0.088	0.088
4	0.705	0.247	0.243

**Table 5:** Probability profile on state space from ERRR-HMM, volcanic case.

*m*	$h=1$	$h=100$	$h=500$
1	0.005	0.120	0.128
2	0.102	0.383	0.401
3	0.760	0.413	0.378
4	0.132	0.078	0.070

**Figure 4:**
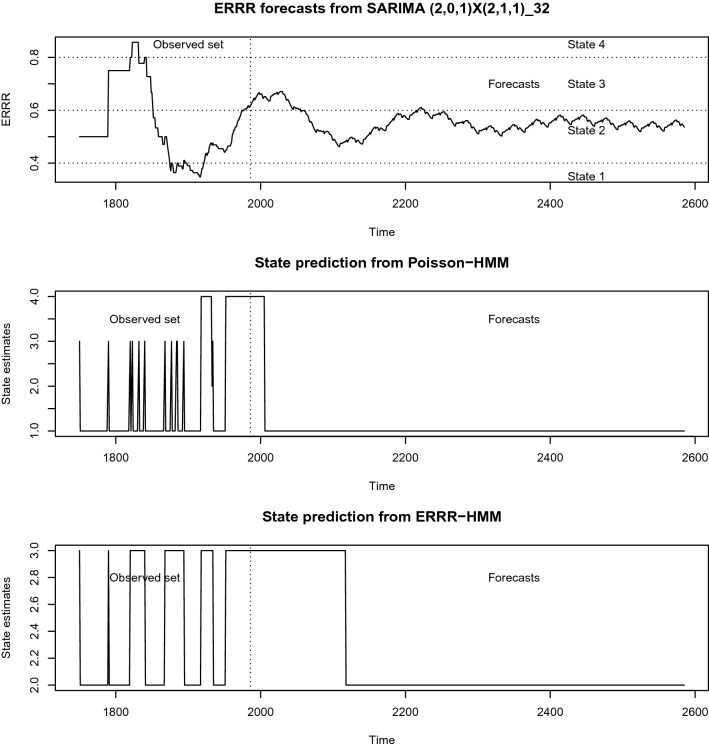
State predictions for volcanic interaction.

Thus, at $h=1$ (i.e., at 1985 + 1 = 1986), the Poisson-HMM estimates the most likely state of the hidden chain to be 4, while the ERRR-HMM predicts it to be 3. The comparisons show that with an increasing forecast horizon, the state distributions gravitate towards the lower states of 1 and 2. Another observation concerns the variation in the highly plausible state estimates. The two most likely states the Poisson-HMM option generates are 1 and 4, spaced wide apart, and this pair stays unchanged regardless of how close ($h=1$) or distant ($h=500$) the forecast distribution is. Those that the ERRR-HMM option gives, however, are not. They transition from states 3 and 4 to states 2 and 3 with the passage of time, with the states being close neighbors in both instances. We varied the forecast horizon sequentially through to 600, chose the most likely states from both models and stored them in the lower two panels of Fig. 4.

It is natural to check next how the observed ERRR series (a discrete version of which was taken as the states that generated observations *in the past*) is likely to propagate in times ahead and whether it is in keeping with the most likely state estimates from the competing models mentioned above. Tan et al.^27^, Ho and Bhaduri^28^, and Ho et al.^1^ have described ways of modeling smoothing statistics like ERR and ERRR through seasonal Autoregressive Integrated Moving Average (SARIMA) models of the Box-Jenkins type. We implement a similar prescription on our current ERRR series with a SARIMA $(2,0,1)\times (2,1,1)_{32}$ model (found through a usual model selection process) and track 600 years’ worth of forecasts in the first panel of Fig. 4. These forecasts mostly oscillate within state 2, which is also the state favored asymptotically by ERRR-HMM. Its competitor Pois-HMM, however, favors the neighboring state 1.

### Forecast distributions through cross-validation

While the previous subsection explains forecasts for the underlying state chain from the two competing models, the present one concentrates on the observable data prediction comparisons. This is crucial since despite the state values $\{ \vec {C_{t}}\}$ and the data values $\{ \vec {X_{t}} \}$ being often correlated, it is frequently the latter that prompts precautions and dictates policy formulations. The history $\{ \vec {X_{t}} \}$ can be utilized once again as Zuchchini and MacDonald^44^ demonstrates, to extract likely future observations through another set of conditional probabilities

23 $$\begin{aligned} P(X_{T+h}=x|\vec {X^{(T)}} = \vec {x^{(T)}}) = \sum \limits _{i=1}^m \epsilon _{i}(h) p_{i} (x), \end{aligned}$$

where $\epsilon _{i} (h)$ is the $i{\text{th}}$ entry of the vector $\vec {\alpha }_{T} /(\vec {\alpha }_{T} 1^{'})$. Again, corresponding to every value of the forecast horizon *h*, (23) generates a probability profile, one that sits on the countably infinite observation space (volcanic or hurricane counts and unbounded, discretely) unlike the ones in the previous section that sat on the finite state space. At this juncture, we employ a cross-validation study. This is typical of several modeling approaches where one wants to ensure reasonable predictive power with observed data pretended to be from the future, prior to implementing the model in practice. With the volcanic example, for instance, we partition the 236 years’ worth of observations into two disjoint blocks—the first 216 to build our models with and the last 20 to test them on. The mean parameters $\{ \vec {\lambda }\}$ and the t.p.m. $\Gamma$ were re-estimated using the training observations and the probability profiles are shown in Tables 6 and 7 for different choices of *h*.

**Table 6:** Probability profile on observation space from Poisson-HMM, volcanic case.

*x*	$h=1$	$h=3$	$h=5$
0	0.507	0.523	0.566
1	0.308	0.298	0.271
2	0.134	0.130	0.118
3	0.039	0.038	0.035
4	0.008	0.008	0.008
5	0.001	0.001	0.001

**Table 7:** Probability profile on observation space from ERRR-HMM, volcanic case.

*x*	$h=1$	$h=3$	$h=5$
0	0.408	0.422	0.437
1	0.361	0.352	0.344
2	0.165	0.159	0.155
3	0.050	0.049	0.047
4	0.012	0.011	0.011
5	0.002	0.002	0.002

Although the number of volcanic eruptions could, in theory, be any large number, we have observed that the probabilities from both models tail off rapidly beyond five eruptions. So, we have truncated Tables 6 and 7 at $x=5$. A look at the original time series or the ERRR curve (left panel of Fig. 1) convinces one of the increased eruption tendencies of Kilauea over the last two decades. The probabilities shown in Tables 6 and 7 demonstrate that ERRR-HMM is better than Poisson-HMM in detecting this fact—the higher counts are given higher probabilities by the former for every choice of the forecast horizon. To elaborate, we may focus on $h=3$. Translated to real time, this horizon stands for 1965 + 3 = 1968 (the period 1750–1965 was used as the training set). During 1968, Kilauea erupted twice. The established method Poisson-HMM gives this event a probability of 13% while our proposal ERRR-HMM makes it more likely with 16%. Similar analyses were carried out with the “Strong–Weak” hurricane interaction with probabilities described in Tables 8 and 9.

**Table 8:** Probability profile on observation space from Poisson-HMM, hurricane case.

*x*	$h=1$	$h=3$	$h=5$
0	0.059	0.058	0.058
1	0.156	0.154	0.153
2	0.211	0.210	0.209
3	0.199	0.198	0.198
4	0.150	0.150	0.151
5	0.098	0.099	0.100
6	0.059	0.060	0.061
7	0.034	0.035	0.035
8	0.018	0.019	0.019
9	0.009	0.009	0.010
10	0.004	0.004	0.005
11	0.001	0.002	0.002
12	0.000	0.001	0.001

**Table 9:** Probability profile on observation space from ERRR-HMM, hurricane case.

*x*	$h=1$	$h=3$	$h=5$
0	0.050	0.047	0.046
1	0.134	0.129	0.126
2	0.190	0.184	0.181
3	0.190	0.187	0.186
4	0.155	0.156	0.157
5	0.112	0.115	0.117
6	0.074	0.078	0.080
7	0.046	0.048	0.049
8	0.026	0.028	0.029
9	0.013	0.014	0.015
10	0.006	0.007	0.007
11	0.003	0.003	0.003
12	0.001	0.001	0.001

These profiles are truncated at twelve observations due to negligible probabilities beyond this point. With the cross validation exercise on the volcanic example rendering confidence in the forecasts through a check with *made up* future observations, the forecast distributions for the hurricane interaction are made using the entire available data set. The models may next be set to predict observations that happen in the *real* future. Again, we draw attention to a few interesting special cases. During both $h=3$ (i.e., 2013 + 3 = 2016) and $h=5$ (i.e., 2013 + 5 = 2018), the West Atlantic basin saw four strong hurricanes with two in 2018, Hurricane Helene and Hurricane Oscar (each with maximum wind speeds 96 knots) being classified marginally strong (please refer to Table 1). The Poisson-HMM attaches a probability of 15% (Table 8) to this event for 2016 while the ERRR-HMM makes it more likely with 15.6% (Table 9). For 2018, once again, the former option estimated it to be less likely (with 15.1% chance) compared to the latter (with 15.7% chance).

Another observation merits mention. Both models agree on the *trend* of evolving probabilities. This is evidenced both through Tables 6 and 7 for the Kilauea counts where a steadily decaying probability trend is observed with every value of *h* and through Tables 8 and 9 for the strong hurricane counts where, with both models, the estimated probabilities initially inflate and eventually deflate. This similarity aside, the newer option ERRR-HMM seems to predict high data values more accurately than its predecessor. Poisson-HMMs tend to exhaust most of the unit probability density over low *x* values. ERRR-HMMs, however, conserve the density over such ranges and distribute the residue accumulated over the larger *x* values. A partition almost always seems to exist that switches the probabilistic dominance of Poisson-HMMs over ERRR-HMMs. With the Kilauea counts, for instance (Tables 6, 7), $x=1$ is the separator (focusing on $h=1$) below which the probabilities from the Poisson-HMM are larger than those from the ERRR-HMM and above which, the pattern flips. With the strong hurricane counts (Tables 8, 9), $x=3$ plays that role. It may be noted that in the context of natural hazards monitoring, these high data values usually spell disasters—more volcanic eruptions, hurricanes, landslides, earthquakes, etc., and must be predicted accurately. ERRR-HMM has been designed and revealed to achieve this goal.

### One-out conditional distributions

**Figure 5:**
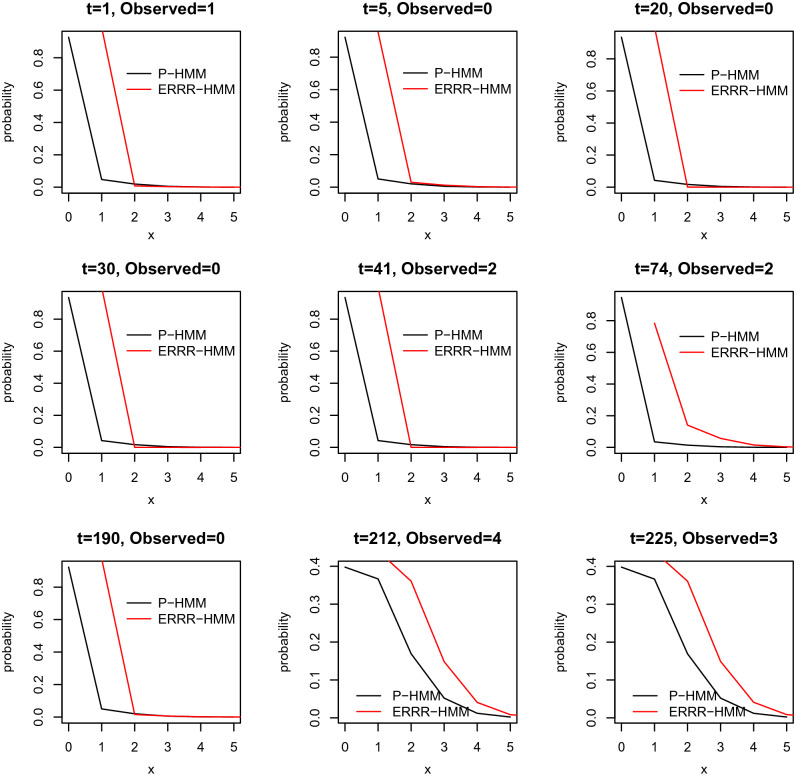
One-out conditional distributions for volcanic interaction.

**Figure 6:**
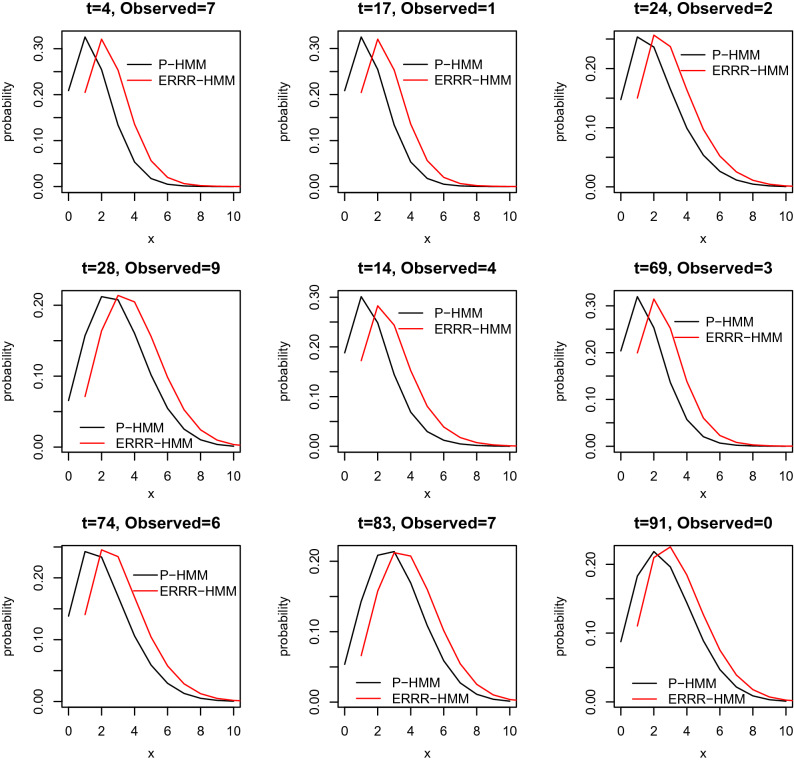
One-out conditional distributions for volcanic interaction.

The next inferential tool we deploy is one where one observation from the history is removed and the remaining are employed to estimate it. With $\vec {X}^{(-t)}:=(X_{1},\ldots ,X_{t-1},X_{t+1},\ldots ,X_{T})$, these conditional densities^44^ are given by:

24 $$\begin{aligned} P(X_{t}=x|\vec {X}^{(-t)}=\vec {x}^{(-t)}) = \sum \limits _{i=1}^m w_{i}(t) p_{i}(x) \end{aligned}$$

where the scales $w_{i} (t)$ are appropriate functions of the observations $\vec {x}^{(-t)}$ and the model parameters. Using (24), we can examine the one-out conditional probability distributions from both the established P-HMM and our proposal, the ERRR-HMM as the deleted time *t* changes. The probabilities have been graphed on Figs. 5 and 6 for the volcanic and the hurricane example respectively.

We explain Fig. 6 here, with similar interpretations holding for 5. The horizontal axis on each subgraph in Fig. 6 shows the number of possible hurricanes (i.e., the observation space *x*) while the vertical axis represents the probability of witnessing that many at the specified time point assuming the availability of the residual history. The actual number observed in each case is also recorded on the top for easy comparisons. For computational convenience, we have set the time origin 1923 as $t=1$. The central panel of the last row (corresponding to time point $t=83$) as an example, reveals that at that at this point in time, the actual number of hurricanes was 7 and ERRR-HMM (shown by the red line) makes this event more plausible, through an elevated probability, than Poisson-HMM. Consistent with our findings in the previous subsection, we notice that our proposal performs better in modeling large counts at instances like $t = 28, 14, 74, 83$, etc. (but not, for instance, $t=91$, when there were no hurricanes)—and those are the years that typically inflict greater damages, and hence necessitate careful forecasting.

### Analysis of pseudo-residuals

A modeling routine usually draws to a close with an analysis of the resulting residuals. This is to ensure the model assumptions have been satisfied. In the present context, it can be shown^44^ that if the underlying model is true,

25 $$\begin{aligned} z_{t}=\Phi ^{-1}(P(X_{t} \le x_{t}|\vec {X^{-t}}=\vec {x^{-t}})) \end{aligned}$$

is a realization of a standard normal variate if the data being modeled happens to be continuous. For discrete counts like our examples, this is checked through a normal pseudo-residual segment $[z_{t}^{-},z_{t}^{+}]$ where

26 $$\begin{aligned} z_{t}^{-}= & {} \Phi ^{-1}(P(X_{t} < x_{t}|\vec {X^{-t}}=\vec {x^{-t}})) \end{aligned}$$

27 $$\begin{aligned} z_{t}^{+}= & {} \Phi ^{-1}(P(X_{t} \le x_{t}|\vec {X^{-t}}=\vec {x^{-t}})) \end{aligned}$$

**Figure 7:**
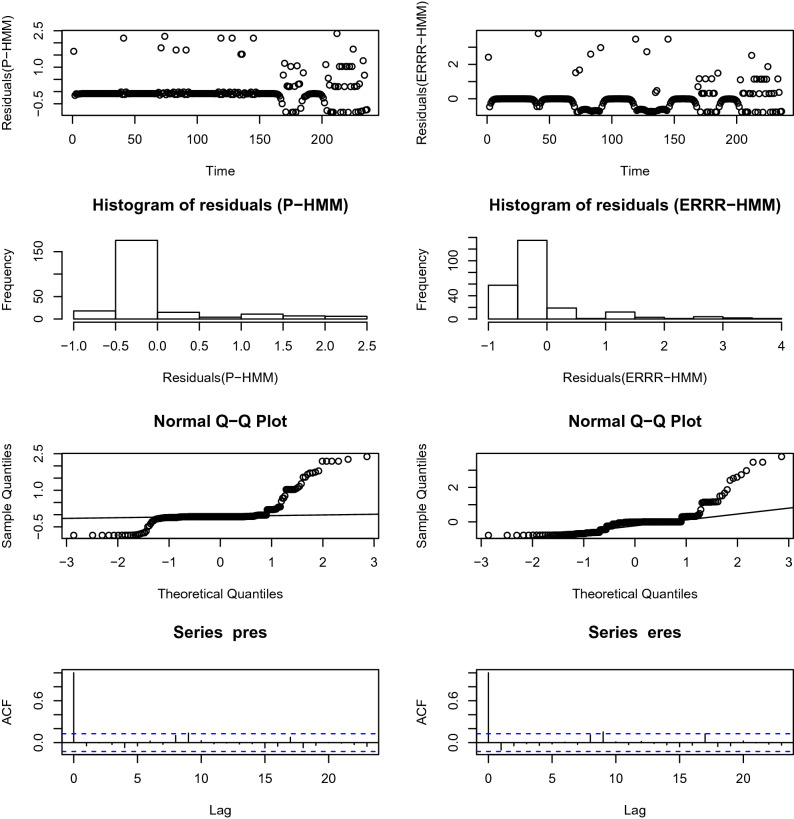
Pseudo-residual analysis for volcano interaction. Left panel—Poisson-HMM, right panel—ERRR-HMM.

**Figure 8:**
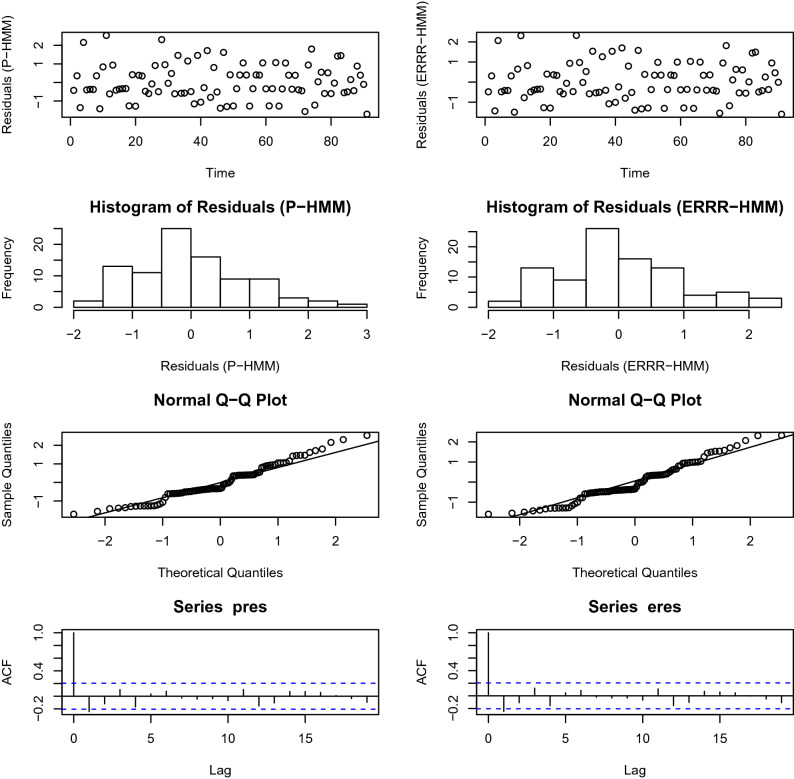
Pseudo-residual analysis for hurricane interaction. Left panel—Poisson-HMM, right panel—ERRR-HMM.

The normality and white noise assumptions being checked are routinely found in statistical texts on regression (for example Montgomery et al.^56^) and will not be repeated here. Instead, we reveal the diagnostics plots in Figs. 7 and 8.

While in the hurricane example, both models furnish adequate diagnostics through normality and uncorrelatedness of errors, in the volcanic example, minor deviations from normality are observed for both competitors. Issues like these may be readily rectified by interested practitioners through several established transformations^56^ on the data values.

## Discussion and conclusions

In a recent work that has generated considerable interest, McPhillips et al.^57^ survey a decade’s worth of academic research spanning diverse disciplines, and seek to formulate a way to define “extreme” events acceptable to all. Scholars from different backgrounds employ different thresholds and conventions. Using text mining, this work found most of the common natural hazards like volcanic eruptions, earthquakes, strong hurricanes, landslides, or floods may generate events that are extreme regardless of the discipline-specific definition employed. Our literature review on available mathematical tools to track these extreme natural hazards shows the relevance of HMMs, or some variants of them, not just in modeling these shocks, but in other disciplines such as speech recognition, bioinformatics, or image retrieval. With the simplest models of this kind, the observations are assumed to be generated by probability distributions dependent on some unobservable states. The state evolution follows the one-step Markov property—the state at any given time depends only on the knowledge of the previous state. A subclass of these models, obtained by setting the state dependent emission probabilities to Poisson, has found popularity with count-based extreme natural hazards like forecasting earthquakes, volcanic eruptions, or landslides, partly because of its theoretical niceties and partly because of its ease of applicability ensured through readily available softwares.

This work notes that if a logical connection binds two hazardous processes evolving over a common time domain, the monitoring of one might exploit the connection through statistically profitable avenues. We substantiate the claim in stages, choosing two examples of stark difference—one from weather science, the other from volcanology, one with a seasonal pattern, the other without, one with a short history, the other long. At the outset, we remind ourselves of the workings of ERRR, a statistic originally developed to understand interaction patterns between the two. Using sound statistical rationales, we subsequently partition the bounded range of ERRR into disjoint blocks and use the discretized ERRR values as the states that generate data to propel one of the two processes being monitored. Information about the interaction, often crucial as the examples demonstrate, is thus incorporated, unlike Poisson-HMM, into the modeling framework through the inclusion of coded ERRRs. We term this technology ERRR-HMM and lay out details on estimating the parameters involved. Unlike the established Poisson-HMM, this estimation is devoid of complex algorithms and uses intuitive relative frequency estimates of ERRR transitions as the probability matrix in the likelihood function. In both the examples surveyed, these estimates are found to be consistent with the ones from Poisson-HMM with the added benefit of *observing* the chain that generates the data, the chain that remains latent (and hence, estimated) in the traditional framework.

We next embarked on comparing the two models using an inferentially exhaustive checklist. Simulations were first extracted using both techniques and compared to the original values. We found that the ERRR-based proposal generates data that conform to the observed pattern at a rate faster than those from Poisson-HMM, especially if the monitored series is of a large length (similar to the Kilauea eruptions). State estimation was conducted next through the process of global decoding and was found to be in fair agreement, with the ERRR-HMM-generated states tracking the observed discretized ERRR better than Poisson-HMM. State predictions for five centuries in the future, conducted next, however, furnished conflicting estimates with the ERRR-based forecasts consistent with some of our time series studies conducted previously. In addition, the states declared to be likely from the newer option are closer in the long run than those from Poisson-HMM.

We next upgraded from the state level to inferences on the observation level. Comparing the actual data values to their probability estimates using cross-validation enriched forecast distributions and one-out conditionals, from both techniques at several time snapshots for each example, we found that ERRR-HMM predicts a period of increased restlessness more accurately than Poisson-HMM. Arguably, such periods pose greater threats and need greater accuracy in their forecasts. ERRR, the key ingredient in our proposed modification, however, renders benefits that transcend mere predictions. In case the ERRR-HMM-generated estimated probability of a large number of Kilauea eruptions for the following year exceeds some threshold, its inhabitants may be relocated. In addition, the wavy pattern of ERRR suggests *where* could they be moved. Its oscillatory behavior indicates Mauna Loa, a close neighbor, is likely to be dormant, and moving Kilauea residents to Mauna Loa will not be expensive owing to their proximity. This thus, aids in minimizing not just loss of lives but also relocation costs. The Poisson-HMM framework, in addition to offering weaker forecasts, cannot render such suggestions about a safe, cost-effective relocation place, owing to its complete divorce from ERRR or similar metrics.

This work may be extended in several meaningful ways. In the inferential exercises investigated, we have let the t.p.m. of the ERRR chain decide its initial (stationary) distribution. If this distribution is forced to coincide with the one generated by the Poisson-HMM, one might have a better agreement between the transition probability estimates. It would be interesting to check how such a switch would modify later inferences, especially the forecast distributions. It is established^58^ that if the Markov chain hidden underneath a stationary HMM is time-reversible, the model as a whole, is so too. For two-state chains similar to the hurricane example, this holds immediately. It is also established^58^ that an irreducible (homogeneous, discrete-time, finite space) Markov chain admits of a positive and unique limiting stationary distribution. Unless the generated ERRR sequence turns out to be monotonic, a non-trivial partition can always be enforced to ensure the discretized chain will be irreducible. Long-term stationary distributions can, therefore, be found. On the applied side, Guo^59^ points out that earthquakes, hurricanes, and floods are typically the gravest natural disasters, inflicting maximum damages. The methodology introduced here may be used to track these other types of hazards. Walter and Amelung^32^ have explored the connection between earthquakes and volcanoes at Mauna Loa. ERRR-based HMMs are general enough to study interactions between two different *types* of hazards too. The numerical computations in this work have been conducted on the statistical software R. Codes may be shared with interested practitioners.

Research into natural hazards monitoring has surged in recent decades at a pace both unprecedented and justified. Newer techniques are being offered, questions are being fine-tuned, cross-disciplinary collaboration and grants are being sought. Workshops like the ones organized by the Natural Hazards Center (https://hazards.colorado.edu/workshop/2019) or Mathematics of Planet Earth (http://mpe.dimacs.rutgers.edu/) are being offered with ever-increasing frequencies. In spite of such increased awareness, recent events like the June 2018 Feugo volcanic eruption in Guatemala claiming 425 lives or the late September Indonesian earthquake-triggered tsunami claiming 2783, establishes the need for relentless and sharper vigilance, the exigency for newer tools and insights. It is this urgency to which our proposal responds, its superiority over an established competitor standing demonstrably valid.
